# Natural product-based pharmacological studies for neurological disorders

**DOI:** 10.3389/fphar.2022.1011740

**Published:** 2022-11-07

**Authors:** Vivek Puri, Neha Kanojia, Ameya Sharma, Kampanart Huanbutta, Divya Dheer, Tanikan Sangnim

**Affiliations:** ^1^ Chitkara School of Pharmacy, Chitkara University, Baddi, Himachal Pradesh, India; ^2^ School of Pharmacy, Eastern Asia University, Rangsit, Pathum Thani, Thailand; ^3^ Faculty of Pharmaceutical Sciences, Burapha University, Muang, Chon Buri, Thailand

**Keywords:** natural products, neurological disorders, clinical research, bioinformatic tools, translational research

## Abstract

Central nervous system (CNS) disorders and diseases are expected to rise sharply in the coming years, partly because of the world’s aging population. Medicines for the treatment of the CNS have not been successfully made. Inadequate knowledge about the brain, pharmacokinetic and dynamic errors in preclinical studies, challenges with clinical trial design, complexity and variety of human brain illnesses, and variations in species are some potential scenarios. Neurodegenerative diseases (NDDs) are multifaceted and lack identifiable etiological components, and the drugs developed to treat them did not meet the requirements of those who anticipated treatments. Therefore, there is a great demand for safe and effective natural therapeutic adjuvants. For the treatment of NDDs and other memory-related problems, many herbal and natural items have been used in the Ayurvedic medical system. Anxiety, depression, Parkinson’s, and Alzheimer’s diseases (AD), as well as a plethora of other neuropsychiatric disorders, may benefit from the use of plant and food-derived chemicals that have antidepressant or antiepileptic properties. We have summarized the present level of knowledge about natural products based on topological evidence, bioinformatics analysis, and translational research in this review. We have also highlighted some clinical research or investigation that will help us select natural products for the treatment of neurological conditions. In the present review, we have explored the potential efficacy of phytoconstituents against neurological diseases. Various evidence-based studies and extensive recent investigations have been included, which will help pharmacologists reduce the progression of neuronal disease.

## 1 Introduction

Information is sent across the body *via* a specialized network of neurons. Neurons use chemical and electrical signals to support the coordination of all fundamental aspects of life. When a neuron releases an electrical or chemical signal, it travels down its axon (a specialized projection) to the neighboring cell. These signals can be retained by root-like dendrites. There are around 86 billion neurons in the human brain. Hence, a growing fetus generates approximately 250,000 neurons each minute (Fields et al., 2020; Heiney et al., 2021). An enormous communication network is created because each neuron is connected to a thousand others. Neurons are the cells that make up the nervous system. Neurons are the cells in the brain responsible for transmitting and receiving signals. Despite their similarities to other types of cells, neurons are characterized by distinct physical and functional properties. Similar to the hundreds of kinds of animals and plants on Earth, thousands of distinct types of neurons exist. Neurons are not all the same in terms of structure, function, or genetics (Duan et al., 2020a; Yang et al., 2020). Neurons are further divided into three categories: sensory (carrying signals from the senses to the CNS), motor (carrying signals from the CNS to muscles), and interneurons (carrying signals from one place to another within the CNS) (Hor et al., 2018; Wan et al., 2018; Smolilo et al., 2019; Duan et al., 2020b). However, neurons come in five distinct varieties. Each exhibits a unique variation on the standard neuron shape.

Brain elements, including cognitive and motor neuron function, can be lost rapidly due to neurodegenerative illnesses, posing a significant problem for the elderly. Alzheimer’s disease (AD), Parkinson’s disease (PD), Huntington’s disease (HD), and amyotrophic lateral sclerosis (ALS) are neurodegenerative illnesses (Angelucci et al., 2019; Jensen et al., 2020). Despite their various clinical manifestations, neurodegenerative symptoms share common traits and mechanisms. Regional cytosolic or nuclear protein aggregation is one of these characteristics (Xu et al., 2021). In AD, extracellular amyloid-beta (Aβ) plaques and intracellular hyperphosphorylated microtubule-binding tau inclusions form (Katsumoto et al., 2019; Roda et al., 2022). Some of the distinguishing features of these diseases are the accumulation of polyglutamine protein aggregates in HD and other repeat CAG-polyglutamine diseases, the intracellular storage of Aβ-synuclein in PD, and the inclusion of TAR DNA-binding protein (TDP)-43 transactive response in ALS, frontotemporal dementia, and other related disorders (Arnold et al., 2013; Toyoshima and Takahashi, 2014). Although a few genetic origins have been found, the primary factor is a complex mixture of genetic and environmental predisposition factors (a balance of hereditary and “sporadic” types in every major neurodegenerative condition). AD is a neurological condition that is the leading cause of dementia among the elderly (Pan et al., 2021a). The amyloid cascade hypothesis proposes that the accumulation of amyloid peptides as fibrils in the human brain is causally related to AD development (Ibrahim and Gabr, 2019). The binding of amyloid-β aggregates to neuronal and non-neuronal plasma membranes causes synaptic and neural network disruption, which is associated with cognitive abnormalities in patients with AD (Hampel et al., 2021). Symptoms include a progressive loss of memory and other cognitive skills as a result of the damage of specific forms of neurons and synapses, which leads to neuronal death (Angelucci et al., 2019). PD is a progressive neurological condition that leads to mortality. It affects 3% of the worldwide population over the age of 60 (Ball et al., 2019). There are two types of PD: familial (inherited in an autosomal dominant or recessive way) and sporadic (idiopathic), which is caused primarily by gene–environment interactions (Halperin and Healey, 2011; Verstraeten et al., 2015; Lill, 2016). Alpha-synuclein (*SNCA*), glucocerebrosidase (*GBA*), leucine-rich repeat kinase 2 (*LRRK2*), vacuolar protein sorting-associated protein 35 (*VPS35*), parkin RBR E3 ubiquitin protein ligase (*PARK2*), and phosphatase and tensin homolog-induced kinase 1 (*PTHIK1*) are the seven genes linked to familial (*PARK7*) (Kruse et al., 2012; Ma et al., 2013; Ankireddy and Kim, 2015; Kalinderi et al., 2016; Mursaleen et al., 2017; Zhao et al., 2018). These genes, as well as particular metabolites and PD-related biomarkers, have been utilized to investigate prospective early detection strategies for PD. The fundamental etiology of idiopathic PD is considered to be gene–environment interactions. Individuals exposed to the same environmental cause are impacted differently, resulting in various illness manifestations (Ball et al., 2019).

## 2 Common targets of neurological disorders

**FIGURE 1:**
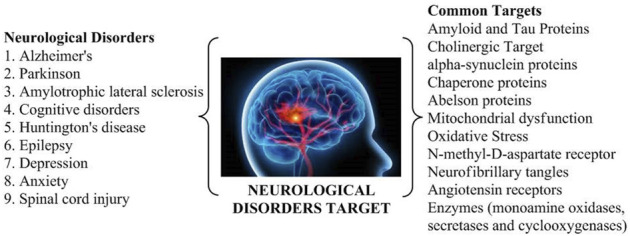
Common targets of various neurological disorders.

The various targets found in neurological conditions (Figure 1) that further can be explored for the drug treatment are mentioned as follows.

### 2.1 Amyloid and tau proteins

The tau and amyloid receptors have been tremendously researched as AD targets (Kent et al., 2020). The main aim is to lower amyloid levels and inhibit amyloid or tau accumulation. Various neuro-proteins, including APOE, APP, BACE (Aβ cleaving enzyme), PS1/2, secretase, and tau, play a key role in the pathogenesis of AD (Chen et al., 2017). Hence, studies are based on the development of novel compounds restricting the aforementioned process for the management of AD.

### 2.2 Cholinergic target

Various research findings have resulted in a facile grasp of the cholinesterase role inside the brain, along with the implementation of cholinesterase inhibitors in the treatment of AD (Stanciu et al., 2020). The further process of the newer generation of acetyl and butyryl cholinesterase inhibitors is being deliberated and scrutinized clinically for AD, resulting in the discovery of antioxidants, hormonal delivery, hypolipidemic compounds, anti-inflammatory drugs, and vaccinations (Santos et al., 2018).

The current study analyzes the common pharmacological targets and biological prospects for current and futuristic natural drugs. Multi-targeted techniques in oxidative stress and neuroinflammatory pathways, along with other target approaches and the extensive role of different phytoconstituents in neurodegenerative diseases (NDDs), are highlighted (Martins et al., 2020).

### 2.3 α-Synuclein protein

A 140-residue protein, presynaptic in the brain and called α-syn, is essential for the movement and synaptic vesicle fusion and controls dopamine (DA) release at presynaptic terminals. In the typical human brain and cerebral spinal fluid (CSF) fluid, α-syn has a physiological concentration of 1 μM and 70 pM, respectively (Domenighetti et al., 2022). When it binds to lipid vesicles, it transforms from its natural state of an unfolded monomer to α-helical conformations (secondary structure). This results in the misfolding and accumulation of α-syn upon destabilization in neurons. The monomeric protein α-syn is inherently disordered and exists in several conformational states. It is important for several vital metabolic pathways and increasing misfolding-related illnesses, most notably neurodegenerative disorders (Fields et al., 2019).

### 2.4 Chaperone proteins

Pharmacological chaperoning is emerging as a viable therapeutic strategy for the management of several disorders linked to single gene mutations. Small molecules known as chaperones attach to proteins, stabilize them against proteolytic breakdown, or guard them against heat denaturation. Additionally, they function similarly to molecular chaperones in aiding or hindering certain protein–protein complexes (Gouda et al., 2022). Several animal models of neurodegeneration have shown that distinct chaperone proteins are neuroprotective. Targeting the cytoplasmic chaperone Hsp90 and, by extension, enhancing the cellular response to stress may constitute a feasible therapeutic strategy for NDDs, although this hypothesis has to be proven and new drugs have to be developed (Lindberg et al., 2015).

### 2.5 Abelson (c-Abl) proteins

Cellular and oxidative stresses activate the protein Abelson (c-Abl), a member of the tyrosine kinase family. It is made up of the SH3, SH2, and catalytic domains. The function of c-Abl depends on where it is located within the cell. c-Abl promotes cellular adhesion with a survival mechanism inside the cytoplasm, but it also induces cell death inside the mitochondria and nucleus (Lindberg et al., 2015). Recent studies revealed that c-Abl is activated in response to amyloid beta fibrils and oxidative stress in AD and PD, as well as in animal models and neuronal cultures (Haron et al., 2021).

### 2.6 Mitochondrial region

It has been discovered that mitochondrial dysfunction is a universal trait of all neurological diseases. It is a major contributor to the onset and advancement of NDDs. Mitochondria play a pivotal role in health and disease by participating in various cellular processes, including maintaining a healthy intracellular Ca^2+^ balance, producing reactive oxygen species (ROS), initiating the intrinsic apoptotic pathway, and synthesizing heme and steroids. Mitochondria also play an important role in neural activity and plasticity and the formation and differentiation of brain cells (Werner and Olanow, 2022). Unusually formed and differentiated neurons emerge from defects in these pathways. Altered signaling of the apoptotic pathway has been linked to neurodegenerative disorders, such as HD, PD, ALS, epilepsy, schizophrenia, multiple sclerosis, neuropathic pain, and AD (Ikawa et al., 2021). Although the relationship between mitochondrial dysfunction and neurodegenerative disease onset and development is still not clearly understood, researchers are exploring treatments that control mitochondrial functioning to reduce neuronal damage and mutant protein aggregation (Jamwal et al., 2021).

### 2.7 Oxidative stress

Oxidative stress is still considered the primary treatment target in NDDs. It is important to investigate the several mechanisms that might considerably restore damage caused by ROS and thus slow or stop the progression of NDDs. The enzyme nicotinamide adenine dinucleotide phosphate oxidase is essential for oxidative stress and is a potential therapeutic target for the treatment of NDDs (Murphy and Hartley, 2018).

### 2.8 NMDA receptors

Neurodegenerative disorders, such as AD and PD, have attracted much attention regarding N-methyl-D-aspartate (NMDA) receptors and their functions in these conditions. Overactivation of NMDA receptors (NMDARs) mediates various elements of synaptic dysfunction in numerous central nervous system (CNS) disorders, prompting a great deal of focus on the development of drugs that can inhibit NMDAR activity (Marí and Colell, 2021; Rahman et al., 2022).

### 2.9 MAO enzyme

As an enzyme, monoamine oxidase (MAO) deaminates monoamines and other proteins. Nervous system diseases, such as AD, PD, ALS, HD, and depression-like disorders, are associated with the large formation of ROS caused by MAO hyperactivation. Although synthetic MAO inhibitors are currently used in clinical practice, they are linked to adverse events such as hepatotoxicity, cheese response, and hypertensive crisis. This has prompted the search for natural MAO inhibitors with a much more excellent safety profile (Gonzalez et al., 2015). The most prevalent neurodegenerative disorders are AD and PD. Based on current research into PD, type B MAO inhibitors, such as selegiline and rasagiline, show highly promising results as neuroprotective medicines. In cellular and animal models, neuronal cells are protected against death by these inhibitors. Stabilizing mitochondria, blocking the death signaling cascade, and activating the pro-survival anti-apoptotic Bcl-2 protein family and neurotrophic factors are all responsible for the neuroprotective actions (Naoi and Maruyama, 2010).

### 2.10 Neurofibrillary tangles

In neurofibrillary tangles, tau, a microtubule-associated protein, has become hyperphosphorylated. An imbalance between the activity of protein kinases and phosphatases acting on tau may occur even before neurofibrillary tangles form because phosphorylated tau proteins accumulate in neurons even before tangles form. To date, no *in vivo* development of neurofibrillary tangles has been observed in experimental models, and the molecular linkage between neurofibrillary tangle and senile plaque formation is poorly known (Mannan et al., 2022).

### 2.11 Angiotensin receptors

The rennin angiotensin system is made up of several different parts, including angiotensinogen, the (pro)renin receptor (PRR), angiotensin-converting enzyme 1 (ACE1), ACE2, angiotensin I (ATI), angiotensin II (ATII), ATII receptor 1 (AT11R), ATII receptor 2 (AT22R), and the Mas receptor (MasR). The rennin angiotensin system plays a crucial role in systemic and cellular pathways to maintain normal blood pressure, fluid balance, and cellular homeostasis. An ACE1/ATII/AT11R axis regulates oxidative stress and neuroinflammation pathways, whereas an ATII/AT22R and/or ACE2/Ang(1–7)/MasR axis enhances neuroprotection pathways. ATII is the primary effector of the RAS, and it exerts its impact by binding to AT11R and AT22R through two competitive arms (Srinivasan et al., 2022a).

### 2.12 COX enzyme

Several research studies have revealed the association between different pro-inflammatory cytokines and PD, and their findings suggest that immunological responses may explain a portion of PD etiology. Evidence supports the hypothesis that cyclooxygenase-2 (COX-2) is over-expressed in mouse models with PD. However, the same research showed that blocking COX-2 reduced the risk of PD by inhibiting the production of potentially harmful DA-quinones (Chinraj and Raman, 2022). Another research revealed that the neuronal cells of PD are severely damaged due to an invasion of T lymphocytes (Brochard et al., 2009).

Memory is a cognitive process in the brain that encodes, stores, and recalls information that has been received. Memory is crucial for learning and communicating with the surroundings (Fuloria et al., 2022). Subjective memory impairment is a frequent finding in adults, although the underlying condition is not detected in most of these patients. Memory impairment (MI) has various etiologies in the absence of physical or psychological disease, including being stressed, feeling ill, feeling melancholy, being exposed to air and noise pollution, adverse effects of certain medicines and substance addiction, and lifestyle factors, such as tobacco use, heavy alcohol consumption, poor physical exercise, and high-fat diet. Memory problems, often known as MI, are important markers for detecting syndromes and their underlying causes. AD, PD, HD, Korsakoff’s syndrome, and Creutzfeldt–Jakob disease are only a few examples (Sarris et al., 2014; Zlotnik and Vansintjan, 2019; Gao et al., 2022). With amnesia and dementia, MI mostly impairs declarative memory; however, this is not necessarily the case with dementia, defined as a decrease in two or more domains of cognition. In other words, dementia not only damages declarative memory but also affects other aspects of memory. Dementia has direct and secondary effects on memory (Vidyanti et al., 2022). Primary memory impairment can involve a deficit in declarative memory, which is one of the cognitive regions affected by AD. Memory capacity is harmed in a secondary case when there are cognitive abnormalities that might limit memory performance, such as attentional deficit (Callahan et al., 2022; Guo et al., 2022). There is currently no proven medication that can completely prevent MI from occurring. In contrast, memory enhancement treatments are critical for preserving a patient’s cognitive function to counteract MI risk factors (Gold and Budson, 2008; Wichansawakun et al., 2022).

## 3 Traditional holistic approach for the management of neurological disorders

Traditional medicines could be an alternative option to cure various neurodegenerative disorders because allopathic treatments are limited and have severe adverse effects. Indian ayurvedic medicine offers several plant-derived compounds that may be useful in future research, especially on neurological disorders. The ayurvedic system provides a holistic approach to managing different polyherbal formulations that act as antioxidants and reduce amyloid deposits and neuroprotective, anti-inflammatory, and immunomodulating compounds that alter neuroendocrine-immune activities, enhance memory, activate neurofunctions, and enhance the quality of life. A balanced lifestyle, good eating habits, socio-psychological support, Rasayanas, and psychotherapies as defined in Ayurveda have been recognized as effective approaches to prevent and treat AD and other neurodegenerative disorders (Rastogi, 2010; Ravikumar and Aittokallio, 2018; Sharma et al., 2018; Rastogi, 2019; Sharma et al., 2022).

Natural products, secondary metabolites, and bioactive molecules derived from plants, animals, and microorganisms are key sources of bioactive molecules that have been turned into disease remedies in many circumstances (Zucchella et al., 2018; Miranda et al., 2019; Ratcliffe et al., 2020). On land and at sea, nature has bestowed surplus resources (natural products) on humans. Natural products play an important role in disease prevention and health promotion for people and animals (Cragg and Newman, 2002; Mantovani et al., 2008; Cragg et al., 2009; Villa and Gerwick, 2010). These natural compounds have been shown to have various biological qualities, including antioxidant, anti-inflammatory, and anti-apoptotic capabilities (Villoslada et al., 2008). Natural products used in numerous preclinical models of neurodegenerative conditions have been further confirmed by *in vitro* and *in vivo* investigations. Phytoconstituents, such as polyphenolic antioxidants, are present in herbs, fruits, nuts, and vegetables, as well as marine and freshwater flora (Aboulwafa et al., 2019; Rehman et al., 2019). These phytoconstituents may help prevent neurodegeneration and improve brain memory and cognitive abilities. They are also thought to play a key role in preventing and treating neurodegenerative illnesses, including AD, epilepsy, and PD (Ratcliffe et al., 2020; Sharifi-Rad et al., 2020; Mendonça-Junior et al., 2021). The plants that show and prove their therapeutic action against neurological diseases are discussed in Table 1.

**TABLE 1:** Different types of plants along with their biological effects.

Plant name/species	Family	Source	Ingredient with biologically significant activity	Action	References
*Ginkgo biloba*	Ginkgoaceae	Leaves	Quercetin, kaempferol, and isorhamnetin	Boosts circulation to the brain	Mashayekh et al. (2021)
*Panax ginseng* C.A. Meyer	Araliaceae	Root and aerial parts	Aglycones, protopanaxadiol, and protopanaxatriol	Neurons survive longer by increasing their supply of survival compounds known as neurotrophic factors	Miranda et al. (2019)
*Scutellaria baicalensis* Georgi	Lamiaceae	Root and aerial parts	Baicalein, baicalin, and wogonin	Protect neurons from oxidative damage	Yoon et al. (2017)
*Curcuma longa*	Zingiberaceae	Rhizome	Curcumin	Inhibition of cytokine production and microglia activation	Yu et al. (2018)
*Vitis vinifera*	Vitaceae	Fruits and seeds	Resveratrol, quercetin, and catechin	Neuroprotective effects	Tabeshpour et al. (2018)
*Salvia officinalis* L.	Lamiaceae	Leaves and flowers	1,8-Cineole, camphor, borneol, caryophyllene, and linalool	Anticholinesterase activity	Kennedy et al. (2006)
*Coffea*	Rubiaceae	Seeds	Caffeine	Acts on adenosine receptors	López-Cruz et al. (2018)
*Camellia sinensis* Kuntze	Theaceae	Leaves	Epigallocatechin, epigallocatechin-3-gallate, myricetin, quercetin, kaempferol, and epicatechin	Antioxidants, protects from oxidative stress, reduces amyloid proteins	Bazyar et al. (2021)
*Bacopa monniera*	Plantaginaceae	Whole plant	Herpestine, d-mannitol, hersaponin, and monnierin	Enhancing neuronal synthesis, kinase activity, restoring synaptic activity, and nerve impulse transmission	Mathur et al. (2016)
*Centella asiatica*	Apiaceae	Leaves	Asiaticoside, brahmoside, brahminoside, asiatic acid, madecassic acid, brahmic acid, isobrahmic acid, and betulic acid	Antioxidant action, acetylcholine esterase inhibitor activity	Hafiz et al. (2020)
*Picrorhiza scrophulariiflora*	Plantaginaceae	Roots	Glycosides, terpenoids, phenylethanoids, glycosides, and phenolic glycosides	Neuritogenic activity	Kumar et al. (2015)

**FIGURE 2:**
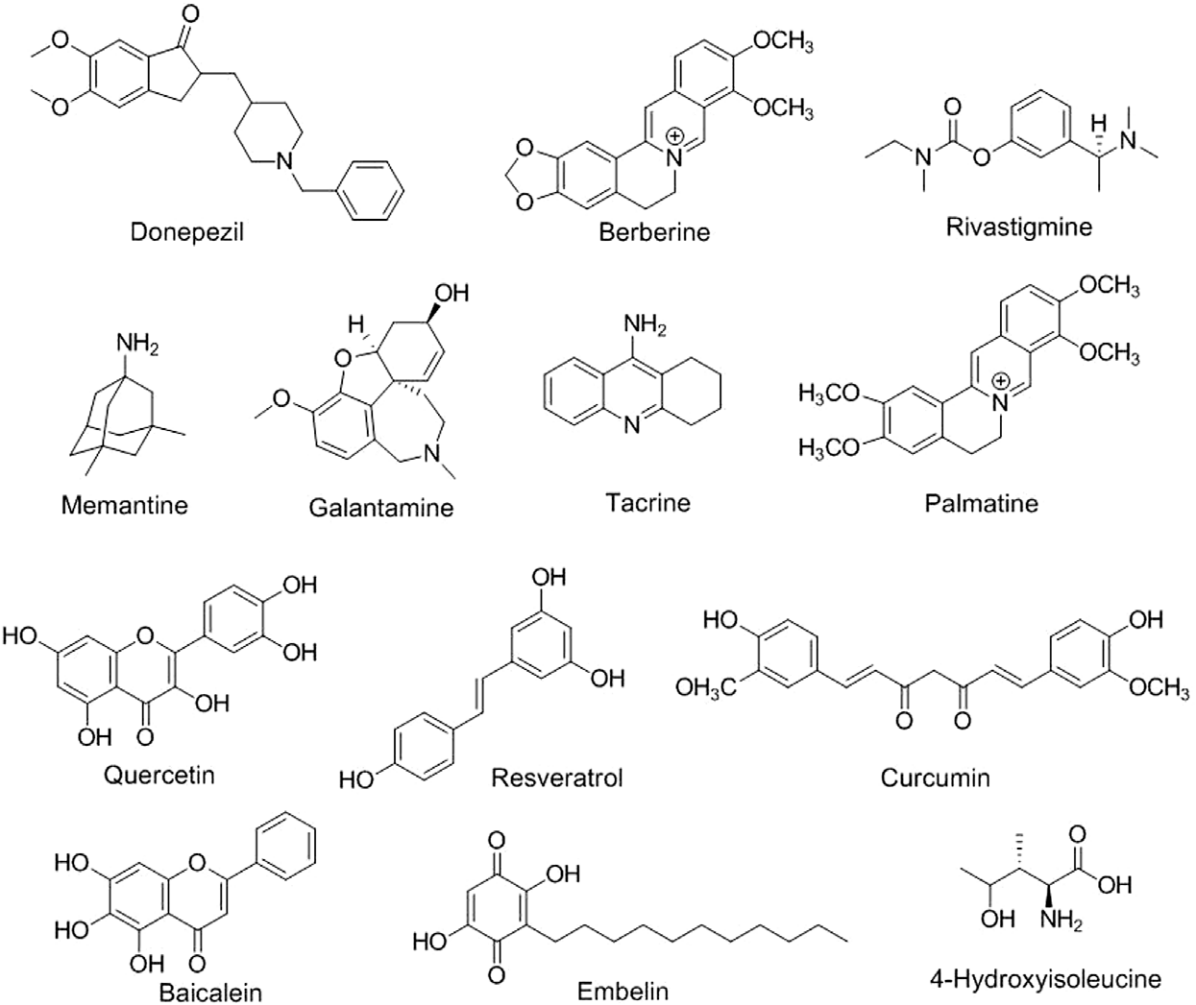
Chemical structures of different phytoconstituents in neurological disorders.

Neuroinformatics is the study of the neurological system *via* the development of databases and tools that aims to design and manage web-accessible databases of experimental and computational data and novel software tools that are necessary for understanding the nervous system in diseased and healthy states (Pu and Li, 2018; Usman et al., 2022). Brain imaging using positron emission tomography (Kaswan et al., 2021; Ruiz-Olazar et al., 2021), functional magnetic resonance imaging (Stefanovski et al., 2021; Li et al., 2022), electroencephalography (Wojcik et al., 2018; Shirbandi et al., 2021), magnetoencephalography (Gorina-Careta et al., 2021), and other methods; several electrophysiological recording methods; and clinical neurological data are examples of neuroinformatics (Sharma et al., 2019). In an interesting study, 679 flavonoid-based compounds and their 481 relative targets were screened, and their bioinformatic analysis exhibited multiple pharmacological pathways, especially for neuronal diseases. Flavone-based targets were remarkably augmented in mitogen-activated protein kinase (MAPK) signaling and neurotrophin signaling pathways, suggesting that natural flavone compounds possess biological effects on neuronal diseases (Qiu et al., 2018; Ravikumar and Aittokallio, 2018). Based on the pattern of substitution of phenyl rings and oxidation and saturation of pyran rings, different modified flavonoid-based compounds can be synthesized, thus exhibiting potent physico-chemical properties and biological activities acceptable for the effective management of neurological-related diseases (Figure 2) (Ayaz et al., 2019).

The concept of medications interacting with many targets has long been seen as undesirable, as it is inevitably related to negative side effects but theoretically can be safer compared to a single-hit target molecule (Hampel et al., 2021). Target-driven approaches often find a poor association between *in vitro* medication effects and *in vivo* effectiveness, thus finding a pivotal research scope. While understanding the underlying pathomechanisms of neurological and psychiatric disorders, searching for new biomarkers, and developing innovative therapies, translational research is one of the most important yet difficult fields for pharmacologists (Wan et al., 2018; Angelucci et al., 2019). Significant progress has been achieved in our understanding of the polygenic, complex, and heterogeneous disease pathways due to the advancement of disease models *in vivo* and *in vitro* (Xu et al., 2021). Diseases that can be studied through translational research include neurodegenerative disorders, such as AD, PD, multiple sclerosis, HD, and ALS, and psychiatric disorders, such as major depressive disorder, bipolar disorder, substance abuse disorder, post-traumatic stress disorder, anxiety disorder, schizophrenia, somatic symptom disorder, autism spectrum disorder, and hyperactive ataxia (Kaswan et al., 2021). There are clinician guides for using neuroscience to guide case framework, understand psychotherapeutic techniques, aid in treatment personalization and outcome prediction, and develop novel mechanistically targeted treatments for disorders (Shirbandi et al., 2021). We extensively added recent and updated key findings and additionally showed the applicability of natural products to improve their appropriate usage in neurological disorders, followed by the incorporation of various clinical studies and patents on phytoconstituents for neuronal diseases. This study focused on assessing various research studies related to the prevention and treatment of NDDs and provided evidence for the efficacy of natural products. It also sparked interest in the development of novel medications for neurological disorders derived from plant sources.

## 4 Phytoconstituents in different neurological disorders

### 4.1 Alzheimer’s disease

AD and dementia are diseases of the elderly society and have become one of the major concerns in health management because of the unattainability of medicinal treatment in this area (Liu et al., 2022). Pathophysiologically, AD is an accelerating neuro-degenerative disease, resulting in the change of behavioral patterns and cognitive defects, and is the recurring source of dementia in approximately 80% of the diseased population, expected to increase three times by 2050 (Zhang et al., 2021a). Various target receptors are responsible for this condition, including the scarcity of important neurotransmitter acetylcholine (ACh), accumulation of β-amyloid proteins, largely phosphorylated tau plaques, and variation in glutamate pathways, neuro-inflammation, and different pathways, which participate in the pathological mechanism of the particular diseased condition (Thomford et al., 2018). In fact, the following are the natural phytoconstituent-based drugs that have been accepted clinically in AD, such as cholinesterase inhibitors (tacrine, galantamine, donepezil, and rivastigmine) and glutamatergic system modulators (memantine). However, they have shown lesser symptomatic effect and hepatotoxicity with tacrine (Joshi et al., 2022).

The important pathological attributes observed in the brains of patients with AD are as follows (Husain et al., 2021):

1) Neuritic plaques containing polymorphous deposits of Aβ, a peptide constructed through the deterioration of Aβ initiators;
2) Neuro-fibrillary tangles, along with the dense irregular bundles inside cytoplasm based in the neuronal system consisting of the modified form of the microtubular-assisted proteins.

The present pharmacological treatment depicts lesser symptomatic positive outcomes. Due to the multi-factorial causes, the advancement of novel molecules is aimed at multi-targeting therapy such as cholinesterase inhibition, anti-amyloid effects, β-secretase and MAO blockage, nitric oxide delivering ability and interactivity with cannabinoid, and NMDA or histamine receptors, contributing to an effective approach in AD. Interestingly, the clinically approved treatment for AD is based on natural phytoconstituents, and its recent developments are described in the following (Jankowska-Kieltyka et al., 2021).

By considering the “single-molecule multiple-target regimen” for the discovery of newer drugs in AD, natural molecules have found dominant interest. Regardless of the less-acknowledged success of synthetic compounds in AD, pharmacokinetics and pharmacodynamics (safety issues) are their crucial restricting steps (Stanciu et al., 2020). Contrarily, natural molecules extracted from herbal, nutritional, or marine origins have shown effectiveness in research studies based on a multi-targeting approach (Ciccone et al., 2021). Among many phytoconstituents, curcumin mitigates cognitive impairment symptoms by modulating inflammatory mechanisms in the brain, decreases free radical burden and metal ion chelation, and blocks Aβ aggregation. Furthermore, has proved to be a favorable candidate for AD and PD. Various flavonoids such as apigenin, luteolin, catechins, gossypetin, and myricetin have also been shown to inhibit Aβ accumulation in AD (Wang et al., 2018). Apigenin can modulate matrix metalloproteinases (MMP)-2 and 9, thus playing a neurodegenerative and neuroinflammatory role, especially in AD. Structure–activity relationship (SAR) research data on flavonoids observed that a catechol ring contributes to an important pharmacophoric moiety in multi-pharmacological activity, including AD. Other products, including alkaloids (huperzine A) and resveratrol, have different biological effects and can interact simultaneously with more than one target of this neurological disorder, showing better effectiveness (Patil et al., 2020; Fantacuzzi et al., 2022).

#### 4.1.1 Berberine

Berberine is a natural compound in which quaternary ammonium salt of isoquinoline alkaloids extracted from different plant species such as *Berberis aquifolium*, *B. vulgaris*, *B. aristata*, *Hydrastis canadensis*, and *Tinospora cordifolia* (Neag et al., 2018)*.* Several pharmacological actions of this compound are mentioned in the literature, such as antioxidant, cholinesterase inhibition, MAO inhibition, and hypocholesterolemic effect, along with fewer gastrointestinal side effects (Akbar et al., 2021). In a recent study, berberine (260 mg/kg, oral) has been reported to reduce Aβ_42_ aggregation and tau hyperphosphorylation through remarkably mitigating endoplasmic reticulum (ER) stress (Wu et al., 2021). Similarly, Liang et al. and group discovered the effect of berberine in 3xTg AD (triple-transgenic AD) mice and observed that protein kinase RNA-like ER kinase/eukaryotic translation initiation factor 2α signal pathway was diminished, further declining Aβ growth and thus improving neuronal functions by mitigating ER and oxidative stress (Liang et al., 2021). In another study, berberine was found to lower MI effects as assessed in a triple-transgenic (3xTg) AD mouse model-based assay. Berberine (100 mg/kg, oral) could simultaneously target autophagic clearance and hyperphosphorylation of tau by regulating the Akt-glycogen synthase pathway (Chen et al., 2020).

#### 4.1.2 Resveratrol

**FIGURE 3:**
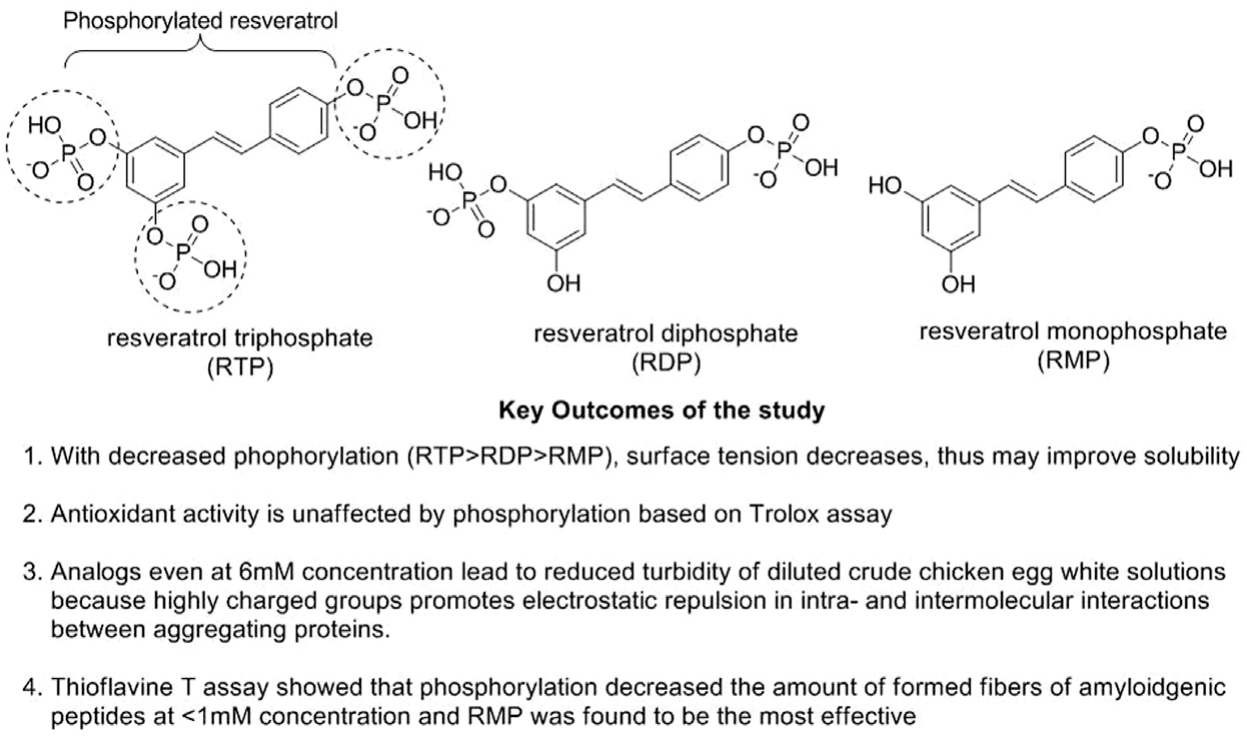
Mechanistic study of phosphorylated resveratrol in AD.

Resveratrol is a polyphenolic compound categorized as stilbenes extracted from plants after exposure to stress, injury, infection (fungal), or UV radiation (Perrone et al., 2017). This phytoconstituent has been reported to have antitumor, anti-inflammatory, cardiovascular, hypoglycemic, and neuro-protective effects with no adverse effects (Zhang et al., 2021b). Resveratrol is readily absorbed in the gastrointestinal lumen, simultaneously exhibiting lesser bioavailability because of its fast metabolism and elimination. Resveratrol plays a significant role in boosting non-amyloidogenic cleavage of the amyloid precursor protein, resulting in advancing the clearance of Aβ peptides and decreasing the degradation of neurons (Sergides et al., 2016). Resveratrol (15, 45, and 135 mg/kg) has been reported to block the cholinesterase effect in AD-based animal assays (Jia et al., 2017). A combination study of melatonin (80 mg/kg) with resveratrol (40 mg/kg) showed that melatonin augmented memory deficit effects in novel object recognition task (NORT) and passive avoidance task (PAT) assays of AD-based mouse models. In contrast, resveratrol enhanced only PAT response in respective animal studies (Jabir et al., 2018). Mehringer et al. explored phosphorylated resveratrol (Figure 3) for their AD-based neuronal properties and observed that these analogs could diminish the accumulation of proteins along with the fibrillation of Aβ42 and insulin based on *in vitro* studies. The *in vivo drosophila* fly model also showed prominent effects with decreased Aβ42 accumulation and enhanced neuroprotective locomotor action (Labban et al., 2021; Mehringer et al., 2022).

#### 4.1.3 Curcumin

**FIGURE 4:**
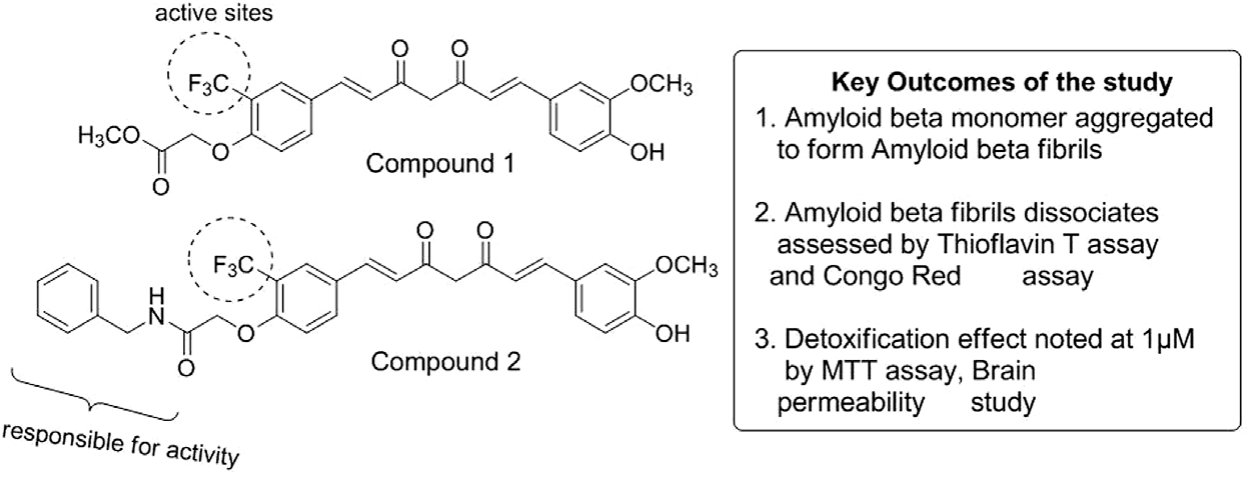
Mechanistic study of curcumin in AD.

Curcumin is the most pivotal turmeric-based curcuminoid and is a popular yellow-colored Indian spice obtained from the rhizome part of *Curcuma longa* and corresponds to the ginger family (Hewlings and Kalman, 2017). Preclinical research has suggested curcumin to prevent or treat many disorders, such as colorectal cancer, cystic fibrosis, and inflammatory and neurological diseases (Jyotirmayee and Mahalik, 2022). Based on phase I clinical data, an oral curcumin dosage of 8,000 mg/day has not resulted in any major adverse effects besides mild nausea and diarrhea. However, excessive usage of this natural compound can harm the gut microbiome, thus obstructing the normal physiological and immunological processes (Gupta et al., 2013). The oral bioavailability of curcumin is relatively low, and many of its metabolites have been detected in plasma after oral intake (Lopresti, 2018). Many recent reviews have assessed the extraordinary role of curcumin in developing tau-focused therapeutics in AD, mainly due to the failure of most of the Aβ-based AD drugs in clinical trials (Sivanantharajah and Mudher, 2022). Current advances have revealed that the phenolic hydroxyl group of curcumin can contribute to the anti-amyloidogenic effect. Phenyl-substituted methoxy groups can show suppression of Aβ42 and APP (amyloid precursor protein), and hydrophobic interactions have also played an amplifying role. Furthermore, the elongation of phenyl rings can have decreased effect in patients with AD (Chainoglou and Hadjipavlou-Litina, 2020). Another systemic analysis carried out the correlation of the 74 target genes of curcumin with AD and experimented through Gene Ontology (GO) mechanism enrichment analysis and Kyoto Encyclopaedia of Genes and Genomes (KEGG). Five important genes were identified using the network pharmacological approach: RARA, APP, PRARG, STAT3, and MAPK1. Computational studies were also carried out to observe that curcumin has a prospective to attach with big active sites of PPARγ, observing better binding scores compared to other protein targets (Vijh et al., 2022). Another molecular docking study showed the molecular modeling studies of curcumin displaying a remarkable binding affinity toward mTOR, TrkB, LXR-β, TLR-2, ER-β, GluN2B, β-secretase, and GSK-3β, which are the critical modifiers of molecular and cellular pathways related to AD (Hannan et al., 2020). Recently, Utomo et al. verified curcumin-based compounds 1 and 2 (Figure 4) in Alzheimer’s *Drosophila* model and observed disassembled Aβ fibrils. The study further showed very low toxicity at 1 µM concentration in N2a cells (neuroblastoma) and prominently recovered its locomotor activity in AD model flies (Utomo et al., 2022).

### 4.2 Parkinson’s disease

The brain motor system is most primarily affected by PD, which causes inflammation and depletion of dopaminergic neurons inside the substantia nigra. A series of factors, including mitochondrial dysfunction, oxidative stress, protein misfolding during synthesis, excitotoxicity caused by different biochemical pathways (such as the glutamate pathway), lysosome impairment, chaperone-mediated autophagy, and the development of Lewy bodies as a result of protein misfolding, contribute to the onset of the disease (Amro et al., 2018). Associated protein (neurofilament) and protein targeting, such as ubiquitinated α-synuclein, are the components of cellular bodies. According to Braak’s staging, Lewy bodies are often located in the olfactory area and the lower part of the brain stem. However, as the illness advances, Lewy bodies also appear in the midbrain (substantia nigra) and forebrain, as well as the neocortex in an advanced stage. The most prevalent classes of phytochemicals with known antiparkinsonian actions include terpenes and numerous subtypes of polyphenols. Alkaloids, carbohydrates, acids (amino and fatty acids), and amides are a few more phytochemical groups containing representatives that have beneficial effects on PD (Lill and Klein, 2017; Zoey et al., 2021). Proinflammatory cytokines, such as prostaglandin E2, interleukin-6 and 1β, and nuclear factor of kappa cells, are reduced in expression, as nuclear and cellular inflammatory signaling, and phytochemicals suppress apoptosis (by reducing either caspases orα-synuclein aggregation), lower dopaminergic neuronal damage, and alleviate DA exhaustion. In order to increase the effectiveness and lower the biological side effects of PD, herbal compounds might be thought of as prospective pharmaceutical medications or as adjuvant therapy, along with traditional therapeutic procedures (Aliakbari et al., 2018).

The striatum has two primary output pathways. 1) The indirect route, which is carried out by inhibiting D2 DA receptors by DA in which the striatum sends GABA-mediated signals toward the neuronal cells in the lateral GP (GPe) and the GPe then sends signals to the STN, which sends glutamate-based excitatory signals to segment (internal) of GP (GPi), as well as SN pars reticulata (SNr). Rigidity and bradykinesia are clinical manifestations of the thalamocortical-spinal route suppressed by GPi and SNr. 2) Simultaneously, the unobstructed path is regulated by DA’s excitatory impact-bearing striatal receptors, and the lack of this neurotransmitter lessens the striatum’s ability to inhibit GPi and SNr (Merzougui et al., 2021).

Although the precise etiology and PD process are still unclear, there has been great progress in understanding the illness’s fundamental mechanisms. This was accomplished by research on genetics, experimental forms of PD, pathological and pharmacological abnormalities of PD, and novel findings on the structural characteristics and physiology of basal ganglia. In this study, we cover the pathophysiology of PD and the natural use of several herbal medicines, as well as their modes of action (Jankovic and Sherer, 2014).

Numerous studies on the use of various herbal remedies and natural items in the treatment of PD have been conducted over the past few years and have been explained below (Ion et al., 2021).

#### 4.2.1 Curcuma longa

In India, *Curcuma longa* is frequently used as a medication for various health issues. It has been established that this plant has anti-inflammatory, antioxidant, chemotherapeutic, anti-proliferative, wound-healing, and antiparasitic properties. Curcumin, the plant’s active polyphenolic component, is assumed to be responsible for these properties (Nebrisi, 2021). Using fibroblasts from patients with PD, who have LRRK2 mutation, as well as healthy controls, curcumin is an effective treatment to address mitochondrial dysfunction in the condition. While post-curcumin treatment showed little impact, pre-curcumin treatment enhanced maximum and ATP-associated respiration. These findings are significant for the therapeutic use of curcumin because they suggest that it would be the most advantageous pre-treatment to toxin exposure. PD fibroblasts with the LRRK2 mutation and healthy control fibroblasts may benefit from pre-treatment with curcumin to prevent mitochondrial damage (Abrahams et al., 2021). Nerve regeneration and anti-apoptotic effects are considerably aided by phosphatidylinositol-3-kinase (PI3k)/protein kinase B (Akt) signaling mechanism and abrineurin pathway. According to recent studies, curcumin regulates the above-mentioned signaling pathways in neurodegenerative disease, positively affecting neuroprotection (Jin et al., 2022).

#### 4.2.2 Resveratrol

Resveratrol, a natural polyphenol, is present in different plant species of grapes and berries. In PD etiology, altered PGC-1 activity and transcriptional dysregulation of its target genes were demonstrated by a recent study, suggesting that PGC-1 may represent a new target for therapeutic intervention. Resveratrol has been reported to increase mitochondrial action by activating multiple metabolic sensors, which in turn activates PGC-α. In addition, the resveratrol administration led to an uptick in the complex I and citrate synthase activity, a reduction in lactate content, an increase in baseline oxygen consumption, and the synthesis of mitochondrial ATP (Katila et al., 2022). These changes supported the transition from glycolytic to oxidative metabolism. Additionally, resveratrol administration increased macro-autophagic flux by activating a mechanism unrelated to LC3. The findings on PD fibroblasts from patients with early onset implied that resveratrol may have potential clinical use in some PD patients. In a different study, Su et al. investigated transgenic and chemically generated mouse PD models, including those caused by MPTP, rotenone, 6-OHDA, paraquat, and maneb (Su et al., 2021). Resveratrol’s neuroprotective effects were mostly focused on reducing oxidative stress and inflammation and improving mitochondrial dysfunction and motor function. Resveratrol also inhibits the production of the enlargement of mitochondria along with the compaction of chromatin and prevents the enlargement of mitochondria and condensation of chromatin (George et al., 2019).

#### 4.2.3 Quercetin

Quercetin, a flavonol-type flavonoid, is present in several fruits and vegetables and is identified as a complementary treatment for PD. The neuroprotective action of quercetin is directly linked with its antioxidant activity, besides stimulating cellular defense against oxidative stress. Additional associated pathways are activating sirtuins (SIRT1) and stimulating autophagy, besides the induction of Nrf2-ARE and paraoxonase 2 (PON2) (Grewal et al., 2021). In another investigation by Josiah et al., the animal studies observed the promising efficacy of quercetin on NF-κB and IκKB gene expressions compared to the rotenone group only. Different research data have exhibited the potential of quercetin for PD by relieving oxidative stress, observing dopaminergic breakdown, and altering neuroinflammation, along with apoptosis (Josiah et al., 2022).

#### 4.2.4 Walnut

The water extract of walnut (*Juglandis semen*) has exhibited pivotal neuroprotective action in various research studies. This extract was found to deplete ROS and NO (nitric oxide) growth, further blocking the loss of DA, thus showing exceptional recovery in patients with PD (Esselun et al., 2022). In another investigation by Yang et al., the walnut-derived polypeptide (TW-7) observed antioxidant action simultaneously initiating autophagy. They further investigated that TW-7 restricted the mitochondrial apoptosis through downregulation of the cytoplasmic cytochrome C, caspase-9, and cleaved-caspase-3 expression (Yang et al., 2022).

#### 4.2.5 Olive leaves extract

Derivatives are isolated from olive leaves, including phenolic compounds, such as hydroxytyrosol, and flavonoids, such as luteolin, apigenin, and apigenin-7-O-glucoside, and their wide range of pharmacological activities, including several properties, such as neuroprotective, antioxidative, antibacterial, antiviral, anti-obese, and anti-inflammatory. The phenolic compounds isolated from olive lowered the syndrome (metabolic) associated with PD (Hadrich et al., 2022).

#### 4.2.6 Myricitrin

Myricitrin, a naturally originated phenolic compound with antioxidant and anti-inflammatory properties, is also known as myricetin-3-O-rhamnoside. Myricitrin’s therapeutic potential was examined in a mouse brain model by Banerjee et al. In the mouse brain, myricitrin reduced MAO activity and increased DA levels. In the PD mouse model, myricitrin could lessen motor incoordination and elevate the DA levels in the striatum (Banerjee et al., 2022).

#### 4.2.7 Baicalein

Baicalein is an active constituent in which *Scutellaria baicalensis* is its natural source. The alcohol extract of *Scutellaria baicalensis* has been reported to decrease nitric oxide (NO) and COX-2 levels (Jeong et al., 2011). This compound also restricts the accumulation of ROS, ATP degradation, apoptosis, and mitochondrial disruption based on rotenone-generated neuronal toxicity (PC12 cells) (Li et al., 2012). Zhao et al. showed that baicalein-treated mice exhibited lower depression-based symptoms after a monthly treatment, and its repeated usage induced α-synuclein dissociation, neuroinflammation blockage, and regulating the homeostasis of neurotransmitters (Zhao et al., 2021). In another study, Song et al. investigated that baicalein can also inhibit the MAO enzyme, and its blocking action on oxidative stress is governed by ERK inhibition in PD (Song et al., 2021; Xu et al., 2022).

#### 4.2.8 Glycyrrhizin

The primary active component of licorice roots and rhizomes (*Glycyrrhiza glabra* L*.*) is glycyrrhizin, which is typically used to treat inflammatory illnesses or even as a tonifying herbal remedy. Ren et al. reported inhibition of the degeneration of DA neurons, reduction of the count of apoptotic cells in the zebrafish brain, prevention of the loss of their vasculature as well as disordered vasculature, and suppression of the locomotor impairment to exert an anti-PD effect on MPTP-induced PD in zebrafish (Ren et al., 2022).

#### 4.2.9 Chicoric acid

A polyphenolic acid called chicoric acid (CA), which is derived from the purple coneflower (*Echinacea purpurea*) and chicory, has been promoted as a nutraceutical to fight infections, inflammation, and obesity. Wang et al. showed that oral pretreatments of CA significantly prevented the motor dysregulation and death of nigrostriatal dopaminergic neurons exacerbated by 1-methyl-4-phenyl-1,2,3,6-tetrahydropyridine (MPTP), including the inhibition of glial hyperactivation and the increase in striatal neurotrophins. It may be inferred that CA showed neuroprotective effects on mice with MPTP-induced PD. These benefits may have been caused by altering the gut microbiota and reducing inflammation along the brain–gut axis (Wang et al., 2021).

### 4.3 Amyotrophic lateral sclerosis

There are broadly two types of ALS: sporadic and familial types. The family variety (5%–10%) has a genetic component but is genetically inherited, whereas the irregular type, which is prevalent (90%–95%), is not inherited. Various neurological conditions, including ALS, are characterized by the degeneration of both motor neurons (upper and lower). Intraneuronal protein aggregates, including protein TAR DNA-binding, superoxide dismutase, and fused in sarcoma, may interrupt normal protein homeostasis and cause ALS and cellular stress (Chandran et al., 2022). These proteins have been thoroughly discovered in ALS animal models and pathological examinations of individuals. Muscle twitching, cramping, soreness, and weakness are static analyses of ALS. Patients eventually develop dysphagia (difficulty swallowing), dysarthria (difficulty speaking), and dyspnea (difficulty breathing) in the advanced stage of the disease. Diet and environmental toxins have also been researched for their links to ALS. For ALS treatment, multidisciplinary methods are reported to be beneficial (Kim and Taylor, 2017; Anakor et al., 2022).

#### 4.3.1 Mecasin

Mecasin, traditional medicine that originated in India, has been shown to have various biological effects *in vivo* and *in vitro*. It also possesses anti-inflammatory properties based on previous investigations and has been discovered for ALS by Kim et al. Mecasin was found to lessen symptom development without causing significant side effects, and the long-term effects of the drug are currently being studied in a phase IIb clinically (Kim et al., 2022).

#### 4.3.2 Morin

It is possible to isolate the yellow chemical component known as morin from the leaves of *Psidium guajava*, *Maclura pomifera*, and *Maclura tinctoria*. Srinivasan et al. studied the effectiveness of flavonoids against amyloids, such as morin, myricetin, and epigallocatechin gallate. Additionally, it was determined that morin has a significant therapeutic potential for developing extremely effective inhibitors for reducing deadly and incurable ALS (Srinivasan et al., 2022b).

#### 4.3.3 4-Hydroxyisoleucine

The insulin sensitivity of rodents is improved by the bioactive amino acid (4-hydroxyisoleucine, HI) extracted from *Trigonella foenum-graecum*. This study focused on brain IGF1/GLP-1 activation, and a study evaluating adult Wistar rats with ALS-like signs found that 4-HI had neuroprotective properties that had been treated with methyl mercury (MeHg^+^). Additionally, evidence points to the neuroprotective advantages of 4-HI in minimizing MeHg^+^-induced behavioral changes, chemical alteration in neurons, and histological impairments in ALS in rats exposed to methylmercury (Shandilya et al., 2022).

### 4.4 Huntington’s disease

The neurological abnormality known as HD is inherited in an autosomal dominant manner and is monogenic. Patients and their families find the illness state traumatizing due to its inheritance pattern (autosomal dominant), progressive nature, and mix of physical, cognitive, and behavioral deficits (Lum et al., 2021). HD is a pathological condition caused by an enlarged CAG trinucleotide repeat in the gene (HTT5) on the chromosome (Yang et al., 2020), which codes for aberrant huntingtin, a potentially pathogenic protein with several functions. The enlarged CAG repeat seen in the mutant protein’s unique polyglutamine pattern is recognized to be hazardous and causes the death or malfunction of neuronal cells (Träger et al., 2015). The striatum neurons are vulnerable to this mutant protein, although HD has been shown to affect the whole brain and body. Exon 1 of the mutant huntingtin protein directly affects transport (axonal), homeostasis (protein), and mitochondrial functioning. The mutant protein’s propensity to aggregate also directly affects these processes. Abnormal huntingtin protein causes neuronal death through several methods. The alternative theory links HD’s neuronal damage to neurotrophic factor losses, glutamate excitotoxicity, and toxic consequences of repetitive associated non-ATG translation mechanisms (Kay et al., 2015).

Memory loss and motor loss of coordination caused by 3-nitropropionic (3-NP) acid were greatly reduced by natural precursors. Reduced lipid peroxidation, enhanced endogenous antioxidants enzymatically, decreased activity (acetylcholinesterase), and increased mitochondrial generation have significantly reduced biochemical changes. Interestingly, 3-NP-induced damage to the striatum was lessened after therapy with certain natural ingredients, as seen by histology. Overall, antioxidant and anti-inflammatory characteristics, maintenance of mitochondrial function, suppression of apoptosis, and activation of autophagy in natural products provided varied levels of neuroprotection throughout preclinical trials of HD (Lum et al., 2021).

#### 4.4.1 Embelin

**FIGURE 5:**
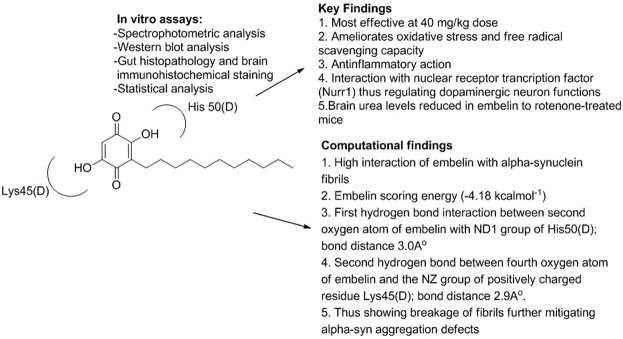
Mechanistic study of embelin in PD and Huntington’s disease.

Embelin’s ability to fortify neurons against 3-NP-induced exploratory HD in rats was examined by Dhadde et al. in which vehicle/embelin was pretreated in adult Wistar rats (doses of 10 and 20 mg/kg p.o.) for a week. Furthermore, embelin significantly reversed behavioral changes, improved antioxidant status, and repaired striatal neuronal damage brought on by 3-nitropropionic acid (Kundap et al., 2017). In an interesting study, embelin and levodopa were analyzed for PD and HD animal studies, which were shown to mitigate oxidative and neuroinflammatory stress. Tyrosine hydroxylase and Nurr1 protein levels were significantly recovered. *In silico* computational studies between embelin and α-syn fibrils were also demonstrated, which validated the strong affinity of embelin approaching α-syn with the help of hydrogen bonding with Lys45(D) and His50(D) residues of α-syn (Figure 5) (Ramachandra et al., 2022).

#### 4.4.2 Curcumin

In India, *Curcuma longa* is frequently used as a medication for several health issues. It has been established that this plant has several properties and is a potential candidate for antioxidant, anti-inflammatory, wound healing, chemical, therapeutic, anti-proliferative, and antiparasitic properties (Mohammadi et al., 2022). Curcumin, the plant’s active polyphenolic component, is assumed to be responsible for these properties. Curcumin’s effectiveness was examined by Aditi et al. in a *Drosophila* model of HD. The injection of curcumin was observed to increase locomotor performance and enhance lifespan in HD flies with advanced illness stages and reduce high reactive oxygen species levels in adult adipose tissue of sick flies (Aditi et al., 2022). The effectiveness of melatonin and curcumin in avoiding the motor deficit and disordered eclosion behavior in the *Drosophila* model of HD was examined by Khyati et al. It can also be deduced that melatonin (100 μg) and curcumin dramatically enhanced the abilities of HD flies to move around and behave in an enclosing manner, restoring the 24 h rhythm of mRNA expression of period and timeless to normal (control) levels (KhyatiMalik et al., 2021).

#### 4.4.3 Lactuca sativa

Malik et al. produced extracts (ethanolic) of the leaves of three different Lactuca sativa (LS) cultivars and assimilated them using HPLC according to their quercetin concentration. The extract with the highest activity level was progressively separated in increasing polarity employing organic solvents (hexane, ethyl acetate, and n-butanol) and an aqueous solvent. It was further concluded that improved behavioral and biochemical indicators demonstrated the greatest reduction of 3-NP-induced HD-like symptoms (Malik et al., 2022).

#### 4.4.4 Baicalein

The neuropharmacological efficiency of baicalein against QA-induced hypertension was assessed in recent research. In the striatum of HD-induced rats, naturally found baicalein, technically known as 5,6,7 trihydroxy flavone, including *Scutellaria baicalensis* and *Oroxylum indicum* (edible plants), has a stronger neuroprotective effect when administered intraperitoneally in doses of 10 and 30 mg/kg. Further analysis reveals that the neuroprotective effectiveness of baicalein exhibits the advancement of psychological and cognitive alterations spurred on by QA (Purushothaman and Sumathi, 2022).

#### 4.4.5 Ugni molinae berries

Arancibia et al. discovered that extracts (phenolic rich) from *murtilla* berries of the 19-1 genotype significantly decreased peptide (polyglutamine) accumulation amounts, corresponding with the regulation in the expression patterns of proteins, which are related to autophagy and thus promising in HD therapy. Berries were extracted by exhaustive maceration with increasing polarity solvents (Pérez-Arancibia et al., 2021).

### 4.5 Epilepsy

Epilepsy is a neurological disease identified with attacks of altered brain responses, resulting in convulsions and seizures, and has affected around 50 million people worldwide (Pearson-Smith et al., 2017). A series of pharmacological events include cognitive impedance and oxidative stress, further contributing to epilepsy-linked recurrent seizures (Mao et al., 2019). In addition, Mao et al. explored the pharmacological mechanism at the molecular level *via* different redox-related neurological cell death modalities in onset seizures. The group also analyzed ferroptosis, a newly discovered lipid ROS-dependent regulatory cell death, which is likely to be a critical mechanism for unfolding epileptic phenotype (Rho and Boison, 2022).

Epilepsy has been classified broadly into four main components (Goldenberg, 2010):

1) Seizure: partial, generalized, and unknown onset;
2) Epilepsies: partial, generalized, combined generalized, and partial unknown;
3) Epilepsy syndrome: juvenile myoclonic epilepsy and Lennox–Gastaut syndrome;
4) Etiology: structural, genetic, metabolic, infectious, immune, unknown.

Epileptic seizures also arise due to the imbalance in the excitation/inhibition response of decreased GABA receptors and the rise in glutamatergic transmission (Karim et al., 2021). Thus, phytoconstituents maintaining this balance [in between the GABA (brain neurotransmitter) and glutamate and blocking of glutamate receptors] will have an efficacious antiepileptic response compared to allopathic antiepileptic drugs showing major side effects among which impairment (cognitive) is undesirable (Kaur et al., 2021). Natural products have exhibited experimentally encouraging results in animal models based on epilepsy. An interesting study discovered the modifications of GABA, GABA_A_, and GABA_B_ targets in the cerebral cortex of epileptic rats, along with the pharmacological application of *Bacopa monnieri*. This plant variety and bacoside-A reversed epilepsy-associated symptoms exhibiting the diminishing role of GABA receptors in epilepsy recurrence (Mathew et al., 2012).

#### 4.5.1 Cannabidiol

Cannabidiol (a phytocannabinoid) is a natural constituent in the *Cannabis sativa*, also known as cannabis or hemp, comprising 80 different forms. One of the cannabidiol forms was approved as an anti-seizure drug in the United States in 2018 (Ryan, 2020). Cannabidiol has been proved *via* recent studies to exhibit anti-epileptic and anticonvulsant activities in acute animal models of seizures. However, their detailed pharmacological pathways remain under investigation (Devinsky et al., 2014). Gray et al. proposed three different pharmacological targets for cannabidiol, including transient receptor potential vanilloid-1, G protein-coupled receptor-55, and equilibrated nucleoside transporter 1, as this phytoconstituent has an attraction for more than one target resulting in neurological excitation applicable in epilepsy (Gray and Whalley, 2020). Concomitantly, cannabidiol was investigated along with other anticonvulsant drugs for its safety, pharmacokinetics, and drug–drug interaction with the help of double-blinded placebo-controlled trials in the recurrent epilepsies in pediatric patients, not just in the epileptic encephalopathy. Cannabidiol administration was observed to be safe and well-tolerated, and new levothyroxine–cannabidiol interaction was reported (Raucci et al., 2020; Cáceres Guido et al., 2021). The structural modification of cannabidiol phytoconstituent majorly comprises its alkyl side chain and the incorporation of phenolic hydroxyl groups on the propenylcyclohexene moiety. The SAR-based studies on cannabidiol, especially on neurodegenerative disorders, are well-reviewed by various groups. Thus, this phytoconstituent has shown great potential in neuropharmacological action (Morales et al., 2017; Prandi et al., 2018; Yousaf et al., 2022).

#### 4.5.2 Apigenin

Apigenin is a flavonoid with several anti-inflammatory, antioxidant, and neurological effects (Salehi et al., 2019). Apigenin and its derivatives are obtained from several plants, such as fruits, vegetables, nuts, citrus, tea, chamomile, thyme, celery, and celeriac, in their glycosidic form (Ginwala et al., 2019). Shao et al. discovered that apigenin could alleviate myeloperoxidase-related oxidative stress and block the ferroptosis of neurological cells. The study developed a multifunctional brain-imaging fluorescence tool and explicated the role of HClO (endogenous hypochlorite) generation by myeloperoxidase in the physiology of epileptic seizures, thus inventing new antiepileptic agents for the prevention and treatment of epilepsy (Shao et al., 2020). The cognitive deficit, a common symptom in epilepsy, was treated with apigenin. Hashemi et al. concluded the biological role of this phytoconstituent in restoring memory deficiency (apigenin significantly increased the number of living neurons in the hilus), thus showing potent anticonvulsant and neuroprotective action (Hashemi et al., 2019).

### 4.6 Depression

Depression is a neurological condition that affects people of all ages worldwide. It is distinguished by emotional, behavioral, health, cognitive capabilities, and behavioral and sleep patterns (Wang et al., 2007). The family and medical history of the patient, early childhood traumas, brain anatomy, and drug consumption are all key contributing variables. Depression is the main cause of disability and a substantial contribution to illness, according to a new World Health Organization report. Multiple complicated biological processes are involved in the pathophysiology of depression (Duman and Voleti, 2012; Zhang et al., 2019). MAPK and cyclic adenosine phosphate signaling are globally accepted to be connected with depression progression, which has sparked much interest in antidepressant research (Pandey et al., 2013; Ekor, 2014). The traditional medical system, which is based on natural ingredients from numerous sources, provides a framework for several commercial depression treatments (Pan et al., 2021b; Álvarez et al., 2022). Metabolic extracts and metabolites derived from many medicinal plants have been shown to have antidepressant effects. In addition to leaves, flowers, and fruits (powdered or unripe), the metabolic extracts are generated from many plant components, such as stem bark, bulb (powdered), the whole plant (seed), petal (stigma), and rhizome (hypocotyl) (Singh et al., 2003; Fakhri et al., 2021; Ranjbar et al., 2022). Collectively, some researchers carried out antidepressant action or neuroprotective benefits by several methods that target the neurological signaling pathways or molecules responsible for depressive illnesses (Lu et al., 2022; Zarneshan et al., 2022). Natural compounds produced from various parts of the plants with a common mode of action are addressed in Table 2. This mechanism includes MAO (MAO-A and MAO-B) inhibitory activity and interactions with dopaminergic (D2), serotonergic, GABA (gamma-aminobutyric acid), adrenergic (α1), and noradrenergic receptor system interactions (Ekor, 2014).

**TABLE 2:** Different types of plants used for depression.

Botanical name	Family	Plant part	References
*Asparagus racemosus*	Asparagaceae	Roots	Dhingra and Kumar (2007), Singh et al. (2009)
*Bacopa monnieri*	Plantaginaceae	Whole plant	Sairam et al. (2002), Girish et al. (2012), Singh et al. (2014), Speers et al. (2021), Zaazaa et al. (2022)
*Benincasa hispida*	Cucurbitaceae	Fruit and seeds	Dhingra and Joshi (2012), Rapaka et al. (2021)
*Phyllanthus emblica*	Phyllanthaceae	Fruit	Dhingra et al. (2012), Muzaffar et al. (2022)
*Glycyrrhiza* glabra	Fabaceae	Roots	Dhingra and Sharma (2006), Martins and Brijesh (2018), Singla et al. (2021)
*Tinospora cordifolia*	Menispermaceae	Stem	Dhingra and Goyal, (2008)
*Rhazya stricta* Decne.	Apocynaceae	Leaf	Ali et al. (1998a), Ali et al. (1998b)
*Nardostachys jatamansi*	Caprifoliaceae	Roots and rhizomes	Karkada et al. (2012), Patil et al. (2012), Li et al. (2021)
*Valeriana jatamansi*	Valerianaceae	Roots and rhizomes	Subhan et al. (2010), Sah et al. (2011a), Sah et al. (2011b)
*Ptychopetalum olacoides*	Olacaceae	Roots	Siqueira et al. (2004); Piato et al. (2009)
*Schisandra chinensis*	Schisandraceae	Seed	Viana et al. (2005)
*Siphocamphylus verticillatus*	Campanulaceae	Stem and leaf	Rodrigues et al. (2002)
*Akebia trifoliata*	Lardizabalaceae	Fruit	Zhou et al. (2010), Jin et al. (2012)
*Boophone disticha*	Amaryllidaceae	Bulb	Spector et al. (2006), Lima et al. (2008), Gadaga et al. (2011)
*Allium cepa*	Amaryllidaceae	Bulb	Sakakibara et al. (2008)
*Paeonia lactiflora*	Paeoniaceae	Roots	Mao et al. (2008a), Mao et al. (2008b), Qiu et al. (2013)
*Anemarrhena asphodeloides*	Asparagaceae	Leaf	Ren et al. (2006)
*Piper longum*	Piperaceae	Fruit	Lee et al. (2005), Lee et al. (2008)
*Polygala tenuifolia*	Polygalaceae	Roots	Cheng et al. (2006), Hu et al. (2010), Kimura and Sumiyoshi (2011)
*Glycyrrhiza uralensis*	Fabaceae	Roots	Wang et al. (2008), Zhao et al. (2008), Fan et al. (2012)
*Trigonella foenum-graecum*	Fabaceae	Seed	Gaur et al. (2012), Mahanti et al. (2022)
*Gynochthodes officinalis*	Rubiaceae	Roots	Cui et al. (1995), Zhang et al. (2002)
*Nelumbo nucifera* Gaertn.	Nelumbonaceae	Seed	Tungmunnithum et al. (2022)
*Zingiber officinale* Roscoe	Zingiberaceae	Rhizome	Singh et al. (2012), Sibi and Meera (2013)
*Curcuma longa*	Zingiberaceae	Rhizome	Takemoto et al. (2022), Yu et al. (2022)
*Cullen corylifolium*	Fabaceae	Seeds	Xu et al. (2008), Yi et al. (2008)
*Rhodiola rosea*	Crassulaceae	Roots	Van Diermen et al. (2009), Mannucci et al. (2012)
*Aniba riparia*	Lauraceae	Seed	Lopes et al. (2018), McCarthy et al. (2022)
*Canavalia brasiliensis*	Fabaceae	Stem	Araújo et al. (2018), Abreu et al. (2022)
*Schinus molle*	Anacardiaceae	Leaf	Khan et al. (2018), Zhou et al. (2020)
*Lobelia inflata*	Campanulaceae	Leaf	Subarnas et al. (1992), Subarnas et al. (1993)
*Apocynum venetum*	Apocynaceae	Leaf	Butterweck et al. (2001), Zheng et al. (2013)
*Salvia rosmarinus*	Lamiaceae	Stem and leaf	Machado et al. (2012), Machado et al. (2013), Singla et al. (2017), Bonokwane et al. (2022)
*Crocus sativus*	Iridaceae	Petal and stigma	Hosseinzadeh et al. (2003), Ettehadi et al. (2013), Abu-Izneid et al. (2022)
*Perilla frutescens*	Lamiaceae	Leaf	Nakazawa et al. (2003), Ito et al. (2008), Yi et al. (2013)

### 4.7 Anxiety

Anxiety disorders are common, incapacitating, frequently chronic, and very co-morbid conditions (Saha et al., 2022). Plant-based medications may provide an extra safe and useful option in addition to traditional pharmacotherapies and psychological therapy, which are the front-line techniques. The term “anxiolytics” refers to phytotherapeutic treatments that may be helpful for anxiety disorders. These treatments typically have effects on the GABA system (Sarris, 2007; Sarris and Kavanagh, 2009), either affecting ionic channel transmission through voltage-gated blocking, altering membrane architecture (Greenfield, 2022), or, less frequently, binding to benzodiazepine receptor sites (such as GABA-a) (Awad et al., 2007), inhibiting GABA transaminase or glutamic acid decarboxylase (Rastogi et al., 2016). Preclinical research in this field has been widely explored, especially by nations such as China, India, Brazil, the United States, Spain, and Germany. Over the past several decades, clinical studies have been undertaken on various plant-based medications for different anxiety and mood disorders. Preclinical research is essential because it frequently expands on existing knowledge of the traditional uses of plant medicines and informs possible human applications. Table 3 discusses medicinal plants used in clinical trials for anxiolytic effects.

**TABLE 3:** Various plant species used for anxiety.

Botanical name	Family	Active constituents	Neurochemical pathways	References
*Achillea millefolium*	Asteraceae	Flavonoids, sesquiterpene lactones, and dicaffeoylquinic acids	—	Nemeth and Bernath (2008), Baretta et al. (2012)
*Aloysia polystachya*	Verbenaceae	Thujone carvone	GABA	Mora et al. (2005), Hellion-Ibarrola et al. (2006)
*Abies pindrow*	Pinaceae	Terpenoids, flavonoids, and glycosides	—	Assad et al. (2021)
*Albizia julibrissin*	Fabaceae	Flavonoids and triterpenoid saponins	Serotonin, 5-HT1A	Kim et al. (2004), Jung et al. (2005)
*Bacopa monnieri* (Brahmi)	Plantaginaceae	Bacoside A	ACh, DA, NA, 5-HT	Stough et al. (2001), Calabrese et al. (2008), Charles et al. (2011), Pase et al. (2012)
*Cannabis sativa/indica* (marijuana)	Cannabaceae	Cannabidiol	Cannabinoid	Campos and Guimarães (2008), Resstel et al. (2009), Bergamaschi et al. (2011)
*Citrus aurantium* (bitter orange)	Rutaceae	Volatile oils and flavonoids	GABA	Akhlaghi et al. (2011), Saiyudthong and Marsden (2011)
*Galphimia glauca*	Malpighiaceae	Nor-seco-triterpene (galphimine B)	5-HT	Herrera-Ruiz et al. (2006a), Herrera-Ruiz et al. (2006b), Herrera-Arellano et al. (2007), Jiménez-Ferrer et al. (2011), Herrera-Arellano et al. (2012)
*Apocynum venetum*	Apocynaceae	Flavonoids	GABA and 5-HT	Grundmann et al. (2007), Xie et al. (2007)
*Crocus sativus*	Iridaceae	Safranal, crocin, and picrocrocin	5-HT, NE, DA, GLU, and GABA	Hosseinzadeh and Sadeghnia (2007), Schmidt et al. (2007), Pitsikas et al. (2008), Hosseinzadeh and Noraei (2009), Ghadrdoost et al. (2011)
*Eschscholzia californica*	Papaveraceae	Benzophenanthridine alkaloids	GABA	Rolland et al. (1991), Rolland et al. (2001), Klvana et al. (2006)
*Euphorbia hirta*	Euphorbiaceae	Alkaloids and phenolics	GABA	Lanhers et al. (1990), Anuradha et al. (2008)
*Justicia* spp.	Acanthaceae	Elenoside	GABA	Navarro et al. (2004), Venâncio et al. (2011)
*Leea indica*	Vitaceae	Triterpenoid glycosides, hydrocarbons, and ursolic acid		Srinivasan et al. (2008), Raihan et al. (2011)
*Panax ginseng*	Araliaceae	Triterpenoid saponins (ginsenosides	Monoamines, HPA-axis, and BDNF	Dang et al. (2009), Jiang et al. (2021)
*Ginkgo biloba*	Ginkgoaceae	Ginkgolides	Dopamine, noradrenaline (norepinephrine)	Kuribara et al. (2003), Woelk et al. (2007), Fehske et al. (2009), Yoshitake et al. (2010)
*Passiflora incarnata* (passion flower)	Passifloraceae	Amino acids, chrysin, b-carboline alkaloids, and flavonoids	GABA	Akhondzadeh et al. (2001), Movafegh et al. (2008), Aslanargun et al. (2012)
*Withania somnifera* (ashwagandha)	Solanaceae	Glycowithanolides	GABA	Andrade et al. (2000)
*Valeriana* spp. (valerian)	Caprifoliaceae	Valerenic acid and valepotriates	Adenosine and GABA	Andreatini and Leite (1994), Andreatini et al. (2002), Benke et al. (2009), Nunes and Sousa (2011), Javan Gholiloo et al. (2019)
*Turnera diffusa*	Turneraceae	Flavonoids (apigenin) and essential oils	GABA	Kumar and Sharma (2005), Kumar et al. (2008)

### 4.8 Spinal cord injury

Mechanisms such as multiple cellular and molecular are activated by acute spinal cord injury (SCI). Su et al. inquired how effectively the Jisuikang (JSK), a traditional drug, works as a treatment in a rat model with established SCI. High-performance liquid chromatography in conjunction with photodiode array detection, electrospray ionization-mass spectrometry, and phytochemical fingerprinting of JSK was used. Additionally, JSK seems to target several pathways (biochemical and cellular) to promote functional recovery and enhance the results of SCI (Su et al., 2013; Islam et al., 2022). To evaluate the therapeutic effects of ethanolic extract of *Mucuna pruriens* (MP) in treating SCI, Chandran et al. used the widely researched standardized Multicenter Animal Spinal Cord Injury Study animal model of the contusive spinal cord. Additionally, MP, at equivalent dosages, was found to be very beneficial in reducing inflammation and/or oxidative stress in various disease circumstances (Rastogi, 2014).

## 5 Role of natural products as biomarkers in neuronal diseases

Using biomarkers of neurodegeneration and neuronal dysfunction can enhance the precision of diagnosis, the ability to track disease progression, prognosis, and the efficacy of therapeutic interventions. Neurological biomarkers are present in the CSF but rarely or at undetectable levels in the blood. Different proteins presented in the CSF, such as neurofilament proteins, tau, and tar DNA-binding protein (TDP-43), have been considerably applied markers to monitor the CNS activity (Viswambharan et al., 2017).

Natural substances have rarely been used as biomarkers in neurodegenerative disorders. However, many biomarkers have been utilized to disclose the molecular pathways of plant extracts for the therapy of NDDs. For example, plasma Aβ40 levels were used to detect the effect of curcumin on AD (Hardy and Selkoe, 2002; Baum et al., 2008). Aβ40 belongs to the *βAPP* gene, the first AD susceptibility gene found, which encodes a glycosylated transmembrane protein of 770 amino acids in its longest isoform. The amyloid cascade theory postulates that an increase in the production of the proteins would result from a mutation in the βAPP gene, with more of the protein eventually broken down to produce the poisonous β-amyloid peptides (Aβ) (Huang et al., 2014). Aβ was also used in a *Huperzia serrata* (Chinese herb) study in the treatment of AD. Cholinesterase inhibitor isolated from *Huperzia serrata* was reported to decrease levels of soluble and insoluble β-amyloid and amyloid plaques in AD mice (Ghodsi et al., 2022a; Mitra et al., 2022).

In the case of PD, α-synuclein aggregation has been used as a biomarker in various *in vivo* studies. Basically, α-synuclein gene is most commonly expressed on elongated arm of chromosome 4 and is a characteristic of PD and also leads to faster progression of the disease. They occur in most forms, including the rare early-onset familial form of PD. A study reported that curcumin extract prevented α-synuclein aggregation and fibrillation in animal models of PD (Bakhtiari et al., 2017).

## 6 Role of bioinformatic studies of plant metabolites in neuronal diseases

Several plants have been used in medicine for neuronal diseases since historical times, and some natural extracts have been developed to commercial medical products. The conventional method of the discovery of plant-based pharmaceuticals is frequently time-consuming and costly. The fast development of high-throughput technology has made it difficult for these labor-intensive methods to stay up. Bioinformatics is vital in the era of high-volume, high-throughput data creation in biosciences. In the realm of drug design and discovery, this has typically been the case. However, the potential use of bioinformatics techniques that can harness plant-based knowledge has received little attention so far. Bioinformatics research has benefited medicinal plant research. In medicinal plant research, the application of bioinformatics techniques leads to faster and potentially more cost-effective discoveries of plant-based treatments.

Most bioinformatic studies of plant metabolites in neuronal diseases have focused on flavonoids. Flavonoids are a family of phenolic substances. This group of phenolic substances has been reported to affect neuroprotection in AD (Mohebali et al., 2018; Sharma et al., 2021). Different side chains may considerably impact the biological activities of flavonoid subclasses, according to systematic correlations between fragments of the chemical structure and biological effects. Flavonoids might considerably enhance the pathways of HD and AD compared to other natural plant products. In addition, systemic examination of targets for various flavonoid subclasses revealed that targets such as MAPT, APEX1, and ALDH1A1, which are strongly associated with the nervous system, were considerably enriched in nearly all flavonoid subclasses. In this situation, the flavonoid multimodal therapeutic potential suggests their value in nervous system medication discovery (Qiu et al., 2018).

## 7 Limitations

Therapeutic efficacy in human patients remains uncertain and limited, although natural products or plant extracts with antioxidant activity have shown excellent efficacy in *in vitro* and *in vivo* animal models. This might be attributed in part to the fact that most clinical studies focus on single compounds. In contrast, plant extracts containing a range of secondary metabolites are more commonly investigated in studies preceding clinical trials. The combination of several active components in extracts can have additive or synergistic effects, resulting in enhanced antioxidant or disease-modifying activities. In addition, clinical trials examine a wide range of subjects with various environmental and genetic origins, as well as various illness symptoms and, in some cases, disease stages. It can be interesting to look at specific people or small groups who show substantial improvement rather than the overall importance of the entire participant population to see why some respond to the treatment and others do not. Furthermore, most clinical studies on natural antioxidants (i.e., natural products or plant extracts) have focused on behavioral or cognitive improvements in patients. In contrast, relatively few trials have properly examined molecular signs of sickness or oxidative stress (Pohl and Kong Thoo Lin, 2018).

## 8 Patent overview

Varied medicinal plant species have been explored in neuronal disorders in the conventional system of natural medicines, and interestingly, unknown species are yet to be scientifically explored. The emphasis on research in the field of herbal compounds in neurological disorders expanded after phytoconstituents were used as a basis for the human treatment of several neurological disorders (Table 4). Ravid et al. formulated a combination of *Uncaria rhynchophylla* herb and an antidepressant or anxiolytic drug therapy for treating or preventing anxiety, stress, depression, and/or symptoms. The combinations, therefore, elicit fast on-set responses in patients (Ravid, 2022). Ichim et al. formulated a nutraceutical of green tea extract and/or *Nigella sativa*, pterostilbene, and/or sulforaphane to overcome treatment resistance of the currently used antidepressants (Thomas et al., 2022). Thamaraikanet et al. prepared a phytochemical extract containing indole alkaloids. Camalexin in aldehyde dehydrogenases mediated benomyl-induced PD. The formulation provides a suitable multi-targeted molecule with antioxidant, neuroprotective, and minimal side-effect properties that can be used as an anti-PD drug (Manasa et al., 2022). Sudhakara Sastry et al. formulated a therapeutically effective nano-polyherbal composition comprising herbal extracts, such as *Allium sativum*, *Bacopa monniera*, *Citrus lemon*, *Citrus sinensis*, *Curcuma longa*, *Cyperus rotundus*, *Lycopersicon esculentum* L., *Mucuna pruriens*, *Nardostachys jatamansi*, *Nigella sativa*, *Prunus dulcis*, *Psidium guajava*, *Sesame indicum*, *Vicia faba*, *Vitis vinifera*, *Withania somnifera*, and *Zingiber officinale* using the phytonanoceutics method, thereby enhancing high bio-efficacy fortified in quality. The composition provides an alternative treatment option for subjects suffering from neurological disorders, anxiety, and/or management of related complications without any side effects (AmanchiBala et al., 2021). Mohanty et al. isolated an anticonvulsant drug from *Cucurbita maxima* and tested it in a convulsion-based animal assay. The pre-treatment with this water–alcohol extract was given biweekly and later exposed to induced electroshock seizures at optimized conditions, and it proved to be effective for electroshock-induced convulsions in rats (Kumar and Nagnath, 2021). Kodimule formulated a composition containing chlorogenic acid and sunflower seed extract in AD (Kodimule, 2021). Palkar and Prasad formulated a synergistic mixture of celery-based extract and various pharmaceutical excipients in brain stroke in different ratios (1:0.1 to 1:5) (Palkar and Prasad, 2021). Vaijanath et al. formulated a Wedelolactone Nasal Formulation. This formulation is made for the nasal drug delivery system to achieve its brain bioavailability for treating or preventing seizures or epilepsy (Vaijnath and Suraj, 2019). Chaudhary et al. formulated a water-soluble extract of *Alpinia galanga* for improving mental alertness and sustaining attention in humans (Chaudhary et al., 2021).

**TABLE 4:** List of different patents on different phytoconstituents for neurological disorders.

Patent no.	Invention	Applicant	Date of publication	References
WO/2022/123572	“A combination therapy comprising uncaria for treating anxiety and depression”	The Open University	16.06.2022	Ravid (2022)
US20220175701	“Treatment of major depressive disorder and suicidal ideations through stimulation of hippocampal neurogenesis utilizing plant-based approaches”	Therapeutic Solutions International, Inc.	09.06.2022	Thomas et al. (2022)
IN202141020016	“Phytochemical extract containing indole alkaloid camalexin for management of benomyl-induced Parkinson’s disease”	Dr. Tamilanban Thamaraikani	11.03.2022	Manasa et al. (2022)
IN201941028495	“A synergistic nanopolyherbal formulation for Parkinson’s disease”	Srimaharshi Research Institute of Vedic Technology	22.01.2021	AmanchiBala et al. (2021)
IN202121057739	“Isolation and identification of suitable anticonvulsant drug from *Curcurbita maxima*”	Dr. Pradeep Kumar Mohanty Nagnath Ramrao Kadam	24.12.2021	Kumar and Nagnath (2021)
US20210330627	“Method of using a chlorogenic acid composition for supporting cognitive function”	Vidya Herbs, Inc.	28.10.2021	Kodimule (2021)
WO/2021/084559	“Synergistic nutritional compositions for treating cerebrovascular diseases”	Celagenex Research (India) Pvt. Ltd.	06.05.2021	Palkar and Prasad (2021)
IN201921009898	“Development and evaluation of wedelolactone nasal formulation for antiepileptic activity”	Sathaye Sadhana Vaijanath	18.09.2020	Vaijnath and Suraj (2019)
US20210205400A1	“Formulation containing an extract of *Alpinia galanga*, a process for the preparation thereof, and uses thereof”	Enovate Biolife Pvt. Ltd.	16.03.2021	Chaudhary et al. (2021)

## 9 Clinical research

Recently, clinical trial reports manifested that mild-to-moderate dementia patients have been cured by employing naturally originated therapeutics (Yiannopoulou and Papageorgiou, 2013). Both studies including clinical trials for test scores and randomized trial for 30 weeks placebo study, were restricted due to resulting hepatotoxicity (Alfirevic et al., 2007). Berberine, another phytoconstituent, displayed symptoms including constipation, diarrhea, bloating, and stomach pain in human subjects with type 2 diabetes (Yin et al., 2008). In a short-term study based on resveratrol, its repeated dose revealed no major adverse effects, but nearly 13% of the individuals had a frontal headache as a side effect (Shaito et al., 2020). In another phase III trial, cholinesterase inhibitors, including galantamine, donepezil, and rivastigmine, were observed to have a lesser memory-enhancing effect, and side effects, including vomiting, nausea, diarrhea, sleeplessness, muscular spasm, loss of fatigue, and loss of hunger, were observed in severe AD subjects (https://clinicaltrials.gov/ct2/show/NCT02035982). In recent report findings, the investigated anti-AD drugs have been excluded based on approximately 200 clinical trials because of inefficacy and toxicity (Mo et al., 2018). Amyloid blockers have not been marketed yet, although they undergo clinical testing (Huang et al., 2020). Toxicity has been reported, Commercialization of such drugs is constrained by concerns of toxicity, but scientists are discovering a novel pharmacological entity with natural existence (Cummings et al., 2021). Indeed, the multitargeting approach by natural agents observes enhanced safety and potentially cognitive modulating abilities, thus contributing to remarkable efficacious compounds (Sartori and Singewald, 2019). Many clinical observations are available in the form of case reports or preliminary clinical trials, which provide essential clinical leads for the initiation of any serious clinical trial in the related area on the background of experimental studies. Interestingly, Ghodsi et al. designed a randomized, triple-blind, placebo-controlled study and evaluated curcumin in 30 idiopathic PD patients and 30 placebo groups as an add-on therapy at 80 mg/kg dose for 9 months. The movement disorder society revision of the Unified Parkinson’s Disease Rating Scale (MDS-UPDRS) part-III was *p* = 0.04, exhibiting a significant difference in patient groups, and nausea and vomiting with *p* = 0.25 and gastroesophageal reflux with *p* = 0.42 were side effects (Ghodsi et al., 2022b). Recently, Wang et al. examined a systematic and meta-analysis overview of the accessible preclinical data and plausible mechanisms of baicalein based on *in vivo* PD studies. Twenty different studies were implied, and the data analysis observed that baicalein can enhance neuroprotective action such as instant motor activity (*n* = 5), pole (*n* = 2), rotarod (*n* = 9), apomorphine-induced rotations (*n* = 4), grid (*n* = 2), and tremor (*n* = 2) tests in comparison to control. The study reported multi-signaling pathways, including neurotransmitter modulation, modifying enzyme activity, relieving oxidative stress, blocking protein aggregation, and further restricting apoptosis (Wang et al., 2020). In another retrospective trial, the pharmacological effect of the artisanal oil formulation of cannabidiol was investigated for epilepsy among 108 pediatric populations. The study observed that 39% of patients showed a major decrease in seizures (more than 50%), and 10% showed no seizures. In contrast, 44% patients exhibited a 50% reduction compared to the 33% with only cannabidiol in the group that was observed receiving combination therapy with cannabidiol and clobazam. The overall results exhibited better alertness and enhanced verbal communication in cannabidiol patients in comparison to the cannabidiol and clobazam patient group, which also showed sedation as its side effect (non-statistically significant difference) (Porcari et al., 2018).

There are few and conflicting pharmacological and clinical studies on the effectiveness of traditional Chinese systems and herbal mixtures in AD. Concerns with irreproducibility may result from this incapacity to deal with uncertainty. Consequently, due to their natural occurrence, promising drug delivery to the brain, and lower adverse effects, the complexes of nanoparticles and herbal plants or their constituents called nano-phytomedicine have currently become essential in the progression of novel neuro-therapeutics. Nanotheranostics is a strategy attracting much interest worldwide for the management of neurodegenerative disorders. Nanoformulations are used in management and diagnosis at the same time. Researchers have created a revolutionary nanotheranostic system that reflects the utilization of nanoparticles and expands the potential applications in this field (Bhattacharya et al., 2022). Toward this direction, Noor et al. established curcumin-based intracerebroventricular injection at a sub-diabetogenic dose of streptozotocin for AD. Curcumin ameliorated the behavioral, immunohistochemical, and most of the neurochemical alterations induced by streptozotocin in the hippocampus and cortex portion, thus showing prospects for brain drug delivery. Thuraisingam et al. formulated nanoemulsions containing *Centella asiatica* crude extract to penetrate the blood–brain barrier using the low-energy emulsification method, showing promising results against epilepsy. Junior et al. compared nanoemulsions of curcumin with free curcumin through an experimental model for PD. The study concluded that curcumin-loaded nanoemulsions and free curcumin enhanced motor impairment decreased lipoperoxidation, modified antioxidant protection, and inhibited the formation of complex I (Ramires Júnior et al., 2021; Noor et al., 2022; Thuraisingam et al., 2022).

## 10 Conclusion

In summary, medicinal plants constitute a significant reservoir of various bioactive ingredients. The implementation of effective multi-targeted drugs for the treatment and prevention of various diseases, including neurological disorders, may result from ethnopharmacology-focused studies that provide a scientific basis for the effective dose and promising toxicological effects on the local community. The key insight is that natural products may hold enormous therapeutic potential for varied neurological diseases as conventional treatments, including synthetic medications, only aim to relieve symptoms and are completely inadequate because they cannot arrest the evolution of the diseased condition. However, the uncertainties regarding the effectiveness and efficacy of several natural products present a challenge. A lot still needs to be studied, described, and discovered. The chemical modification of natural phytoconstituents and molecular docking of those compounds may improve the potency and efficacy of natural products. Thus, to improve patient safety and ethical treatment, clinicians must frequently investigate the employability of all products, such as conventional, complementary, and alternative. Furthermore, experts should deliberately begin to increase scientific understanding of the efficacy and safety of natural products, underlining the need for fundamental research to enhance scientific understanding of the fundamental biological mechanisms. The best sources of novel therapeutics and active frameworks are still natural products. When synthetic and biological chemists collaborate on these case studies, novel structures with the potential to treat a range of human diseases can be investigated.

## 11 Future prospectives

Pain associated with neurodevelopmental disorders and neurodegenerative diseases are common, as are conditions which includes Parkinson’s disease (PD), dementia, epilepsy, and neuro infections caused by malnutrition. The pharmacological properties of medicinal plants have been effective in treating various neurological conditions. Although many different types of plants are available globally, only a few have been researched for neurological problems. Therefore, there are several chances for more exploration of botanicals and their bioactive compounds in this field. In recent years, there has been an increase in interest in natural alternative treatments that encourage fast recovery and avoid side effects. The use of natural compounds in alternative and complementary therapies may result in the identification of novel drug lead compounds. The use of natural compounds to treat neurodegenerative illnesses has gradually become a growing industry. In addition to providing a scientific foundation for the ideal dose and potential toxicological effects on the local community, pharmacological studies can aid in the development of even more effective therapeutically multi-targeted natural compounds for the treatment of various neurological disorders.
